# The Fibrinolytic System in Bacterial Sepsis: A Comprehensive Review of Current Assessment Methods

**DOI:** 10.3390/jcm14176055

**Published:** 2025-08-27

**Authors:** Florin Scarlatescu, Ecaterina Scarlatescu, Jecko Thachil, Dana R. Tomescu, Daniela Bartos

**Affiliations:** 1 Department of Neurology, Clinical Emergency Hospital Bucharest, 014461 Bucharest, Romania; scarlatescuflorin1@gmail.com; 2 Department of Internal Medicine, University of Medicine and Pharmacy Carol Davila, 050474 Bucharest, Romania; bartos_daniela@yahoo.co.uk; 3 Department of Anaesthesia and Intensive Care, University of Medicine and Pharmacy Carol Davila, 050474 Bucharest, Romania; danatomescu@gmail.com; 4 Department of Anaesthesia and Intensive Care, Fundeni Clinical Institute, 022328 Bucharest, Romania; 5 Department of Haematology, University of Manchester, Manchester M13 9PL, UK; jecko.thachil@mft.nhs.uk

**Keywords:** fibrinolysis, sepsis, coagulopathy, thromboelastometry, plasminogen activator inhibitor-1, disseminated intravascular coagulation

## Abstract

**Background:** Fibrinolytic impairment is one of the key factors involved in the pathogenesis of hemostasis disturbances in sepsis, significantly contributing to microthrombosis, organ dysfunction, and mortality rates. While hemostatic assessment in sepsis typically focuses on coagulation activation, evaluating fibrinolytic activity remains challenging due to methodological limitations and a lack of standardization of the currently available methods. **Objectives:** This comprehensive review examines current methods for assessing fibrinolytic activity in bacterial sepsis, their clinical applications, strengths and limitations, and future perspectives for improved diagnostic approaches. **Methods:** We conducted a systematic literature search and identified 52 studies that investigated fibrinolysis assessment in adult patients with bacterial sepsis using biomarkers or global tests. Studies included mainly observational cohorts examining various fibrinolytic assessment methods. **Results:** Fibrinolytic shutdown, primarily mediated by the overproduction of plasminogen activator inhibitor-1 (PAI-1), occurs early in sepsis and correlates with disease severity and mortality. Current assessment methods include plasma biomarker measurements (PAI-1, plasmin-antiplasmin complexes, D-dimer), global plasma-based tests (clot lysis time, plasmin generation assays), and whole-blood viscoelastic testing (rotational thromboelastometry, ROTEM; thromboelastography, TEG). Modified viscoelastic tests incorporating tissue plasminogen activators demonstrate enhanced sensitivity for detecting fibrinolytic resistance. Despite efforts, standardization is still limited, and routine clinical implementation has not been achieved yet. **Conclusions:** Fibrinolytic assessment provides important prognostic information in sepsis, despite methodological challenges. The integration of point-of-care viscoelastic testing with modified protocols shows promise for real-time evaluation. Future research should focus on developing standardized, automated assays suitable for routine clinical practice, enabling personalized therapeutic interventions that target fibrinolytic dysfunction in sepsis.

## 1. Introduction

Physiological hemostasis relies on the balance between clot formation and clot breakdown. Clotting is essential for stopping bleeding until the injured blood vessels are healed, and the breakdown of clots is needed for maintaining blood flow while the healing process is ongoing [1,2]. The regulation of both clotting and fibrinolytic systems depends on different activators, cofactors, receptors and inhibitors [2,3]. Bacterial sepsis frequently leads to hemostatic disturbances characterized by increased coagulation activation, along with dysfunctions in anticoagulant and fibrinolytic mechanisms, and is associated with poorer outcomes [4].

Hemostasis assessment has become a routine practice worldwide, primarily through the widespread use of clotting tests like the prothrombin time and activated partial thromboplastin time. The tests for fibrinolytic activity are less widely performed than those used to study clotting [5]. Except for point-of-care tests such as viscoelastic tests (VET) which are widely available and routinely used in clinical practice for coagulation and fibrinolysis assessment, the laboratory testing of fibrinolysis is cumbersome and often available only in specialized laboratories. VET are increasingly used in clinical practice as more convenient assessment tools for the clot dissolution process. However, they might have relevant limitations for fibrinolysis assessment, especially in scenarios with low fibrinolytic activity. As the cellular components of blood are important for both clot formation and fibrinolysis, VET from whole-blood offer an advantage compared to laboratory fibrinolysis testing based in general on plasma. Rotational thromboelastometry (ROTEM) and thromboelastography (TEG) are the most used VET worldwide. With these devices, fibrinolysis activity is quantified by a decrease in clot amplitude after maximum clot firmness (ROTEM) or maximum amplitude (TEG) is reached. With this method, hyperfibrinolysis is easier to diagnose than low fibrinolytic activity. The decrease in clot amplitude after reaching maximum firmness is small or even zero, both in healthy controls and in patients with hypofibrinolysis, during the limited measurement time [6,7]. Platelet-mediated clot retraction also leads to a decrease in clot amplitude after it reaches its maximum value. Therefore, detecting low fibrinolytic activity with VET is challenging. Various methods to assess fibrinolysis based on the measurement of individual fibrinolytic biomarkers or global tests based on plasma, plasma components, or whole-blood were developed [8,9]. However, a reliable method to measure fibrinolytic capacity in cases with low fibrinolytic activation in clinical practice is lacking.

The present review aims to 1. describe the balance between clotting and fibrinolysis, as well as the dynamics of the fibrinolytic process in sepsis, 2. provide an overview of the primary methods used for fibrinolysis assessment in sepsis, summarizing the strengths and limitations of these assays, and 3. discuss future perspectives for improved assessment of fibrinolytic disturbances in sepsis. The purpose of this review is to underscore the crucial role of fibrinolytic activity in bacterial sepsis pathophysiology, as this aspect is often overlooked in clinical practice due to the aforementioned difficulties in diagnosing low fibrinolytic activity. It seeks to draw attention to the importance of assessing fibrinolytic activity in septic patients, highlighting its significance in patient care and outcomes. The hemostatic changes associated with viral infections, extensively studied during the recent COVID-19 pandemic, will not be covered in this review, which focuses solely on bacterial sepsis.

## 2. The Clotting and Fibrinolytic Disturbances Associated with Sepsis

Coagulopathy is frequent, occurring in about one-quarter of septic patients, and associated with increased morbidity and mortality [4,10]. The hemostatic disorder in sepsis is characterized by increased activation of coagulation accompanied by dysfunction of anticoagulant and fibrinolytic mechanisms such that enhanced fibrin formation is followed by impaired fibrin removal, contributing to organ dysfunction [11]. In severe infections, pathogen-associated molecular patterns (PAMPs) and damage-associated molecular patterns (DAMPs), bind to pattern-recognition receptors on immune cells, primarily monocytes and circulating macrophages, inducing the expression of tissue factor (TF) on their surfaces [12]. The increased thrombin generation observed in severe infections and sepsis is primarily due to coagulation activation through the extrinsic pathway, facilitated by the TF expressed on the surfaces of circulating cells and endothelial cells [12,13,14,15]. In addition, several other mechanisms contribute to coagulation activation in sepsis. These include increased activation of platelets and endothelial cells, complement activation, and the release of extracellular vesicles from various cells that express TF and phosphatidylserine on their surfaces [16,17]. Additionally, polyphosphates released from bacteria and platelets, as well as neutrophil extracellular traps (NETs), play a role in septic coagulopathy [17,18,19].

### 2.1. Immunotrombosis

Inflammation activates and enhances the coagulation process, and in turn, the activated coagulation system increases the inflammatory response, with thrombin serving as the central regulator of the interaction between inflammation and coagulation. Thrombin binds to protease-activated receptor-1 (PAR-1), which is expressed on various cells such as monocytes, neutrophils, platelets, and endothelial cells. This binding amplifies both coagulation and inflammatory responses [19,20]. Conversely, the activation of coagulation plays a significant role in immune defense, a concept known as “immunothrombosis” [21]. During immunothrombosis, the coagulation process helps to limit the spread of invading pathogens by trapping them within the blood clots, thereby preventing their dissemination. It also enhances the recruitment of immune cells to the sites of infection and creates specialized compartments that concentrate antimicrobial peptides. This facilitates immune cell interaction with pathogens and enhances their effectiveness [21,22,23]. Therefore, the increased coagulation is beneficial for immune defense early in the course of infection. However, uncontrolled coagulation activation can become detrimental to the host, leading to widespread microvascular thrombosis and organ failure in later stages [23].

### 2.2. Stages of Septic Coagulopathy

The coagulation disorder observed in sepsis is a dynamic process that progresses through several stages, each varying in speed and severity. Initially, coagulation activation occurs alongside the release of PAMPs, DAMPs, and inflammatory mediators, leading to a phase known as ‘non-overt disseminated intravascular coagulation (DIC)’ or ‘sepsis-induced coagulopathy (SIC)’. This is followed by a stage termed “thrombotic DIC,” which is marked by uncontrolled thrombin generation and the dysfunction of anticoagulant and fibrinolytic systems, resulting in micro-thrombosis and subsequent organ dysfunction (overt DIC) [19,24]. In the late phase of overt DIC, the consumption of coagulation factors and platelets leads to hypocoagulability and bleeding complications [24,25]. Often, patients are recognized only in this late stage of overt DIC, as neither the preceding stage of hypercoagulability nor the critical transition point to hypocoagulability can be easily detected through routine coagulation tests [18,24]. However, the recent literature has suggested the use of specific scoring systems for diagnosing early-stage DIC, tailored to the underlying condition, with SIC score recommended for coagulopathy of sepsis [4,24].

### 2.3. Fibrinolysis

Fibrinolysis, much like clotting, is regulated by a variety of activators and inhibitors [26]. Clot dissolution is mainly achieved by plasmin, which is responsible for the cleavage of fibrin into soluble degradation products [1,27]. Most of the plasmin formation occurs when the inactive pro-enzyme plasminogen binds to tissue plasminogen activator (t-PA) on the surface of fibrin, leading to cleavage and activation of plasminogen [28]. The fibrinolytic system can also be activated through urokinase plasminogen activator (u-PA), which also forms a complex with plasminogen, although it has a lower affinity compared to t-PA. u-PA is present in urine and is produced by monocytes, macrophages, and epithelial cells. u-PA acts on plasminogen via the u-PA receptor, independent of fibrin as a cofactor [29]. Both t-PA and u-PA are inhibited by plasminogen activator inhibitors (PAI-1 and PAI-2). Additionally, plasmin is rapidly inactivated in the body by antiplasmin, resulting in the formation of stable complexes known as plasmin-antiplasmin complexes (PAP) [28,30]. Various other factors such as thrombin-activatable fibrinolysis inhibitor (TAFI), activated protein C system, increased plasma levels of nuclear products such as cell-free DNA (CFDNA) and histones, neutrophil elastases and different proteases are involved in the regulation of the fibrinolytic process acting at different stages of the hemostatic process [26,31,32].

### 2.4. Fibrinolysis in Sepsis

In sepsis, the impairment of fibrinolysis, primarily caused by the overproduction of PAI-1, plays a significant role in the development of micro-thrombi and organ failure [11]. Similarly to the clotting process, fibrinolysis is dynamic, and the low fibrinolytic activity does not always occur at the onset of sepsis. Experimental studies in healthy volunteers who received intravenous endotoxin showed an increase in t-PA concentration within the first three hours after the inflammatory response began, which led to heightened fibrinolytic activity. However, this was followed by an increased PAI-1 release, resulting in the inhibition of fibrinolysis after that initial period [33,34]. The regulation of fibrinolysis in sepsis evolves rapidly, and the increased fibrinolytic activity occurring soon after the release of inflammatory mediators often goes unnoticed in septic individuals. This can be a problem since patients with sepsis are typically included in studies during the later phases of their illness. With the exception of experimental studies in healthy volunteers, where the fibrinolytic activation has been captured early after triggering an inflammatory response by the administration of low doses of endotoxin or tumor necrosis factor, clinical research involving septic patients predominantly describe the later stage of fibrinolytic shutdown [35,36,37,38].

The primary regulators of the fibrinolytic process in early sepsis, t-PA, and PAI-1, are released by the endothelium, which is responsible for the initial pro-fibrinolytic response seen in early inflammation and for the fibrinolysis inhibition observed in later stages of sepsis [33,34,39]. The release of PAI-1 is triggered by proinflammatory cytokines, and elevated PAI-1 levels are commonly observed during inflammatory reactions [40]. Besides inhibiting t-PA and u-PA, PAI-1 has other functions resulting in procoagulant effects. These include the inhibition of activated protein C, limiting the formation of thrombin-thrombomodulin complexes by competing with thrombomodulin for thrombin binding, and the formation of procoagulant microparticles [40]. While PAI-1 is widely regarded as the main factor responsible for inhibiting fibrinolysis during sepsis, TAFI also plays a significant role in shutting down fibrinolysis, particularly in the later stages of sepsis [41,42]. In addition to the role of plasminogen activators and fibrinolysis inhibitors, other proteases may become significant in the fibrinolysis process under specific circumstances. During sepsis, highly activated leukocytes release proteolytic enzymes such as leukocyte elastase and cathepsin G [43]. Leukocyte elastase can digest cross-linked fibrin resulting in different cross-linked fibrin degradation products compared to those produced by plasmin [44,45]. The activation of elastase-mediated fibrinolysis occurs to varying extents in other inflammatory conditions, such as cardiac surgery with cardiopulmonary bypass and after major abdominal surgery [46]. In the early stages of sepsis, neutrophil elastase activates an alternative pathway for fibrinolysis which is insufficient to overcome PAI-1-mediated fibrinolytic shutdown [47]. In health, leukocyte elastase does not interfere with clot lysis due to endogenous inhibitors, however in states with high degree of inflammation, leukocyte elastase reaches high plasmatic concentrations leading to different degrees of activation of non-plasmin mediated clot lysis [48]. In septic patients, activated neutrophils release neutrophil extracellular traps (NETs) that contain CFDNA, histones, and neutrophil granular proteins. The primary role of NETs is to trap and kill pathogens; however it is also known that they activate thrombin generation and inhibit fibrinolysis. CFDNA binds simultaneously to fibrin and plasmin forming a ternary complex, preventing the degradation of fibrin by plasmin [49]. In this manner, CFDNA plays a crucial role in inhibiting fibrinolysis in sepsis in conjunction with PAI-1 [49].

Despite the crucial role of fibrinolytic shutdown in the pathophysiology of organ dysfunction associated with sepsis, this diagnosis is rarely established in clinical practice. This is primarily because the assessment of fibrinolytic activity is not commonly conducted in septic patients outside of research settings. Due to the lack of fibrinolysis testing in clinical environments, specific treatments aimed at addressing low fibrinolytic activation in sepsis have not been developed or tested in large-scale trials.

## 3. Assessment of Fibrinolysis in Sepsis

To assess current literature on potential markers for altered fibrinolysis in sepsis, we conducted a literature search in PubMed on 16 June 2025 using the following search terms: “sepsis”, “septic shock”, “fibrinolysis”, “PAI-1”, “plasminogen activator inhibitor”, “thromboelastometry”, “thrombelastography”, “clot lysis” in various combinations. Studies were included if they investigated fibrinolysis using biomarkers or global tests in adult patients with bacterial sepsis. Study designs eligible for inclusion were observational (descriptive, cross-sectional, prospective, comparative or retrospective cohort studies), and randomized controlled trials published in English. Review articles, editorials, meta-analyses, case reports, case series, in vitro studies, publications without an abstract, animal studies, studies including pediatric patients or patients having associated pathologies possible causing altered fibrinolysis (acute liver failure, chronic liver dysfunction, trauma, post-partum bleeding, solid and hematological malignancies) were excluded. Additional eligible studies were identified by searching the reference lists of articles that were fully assessed.

**Figure 1:**
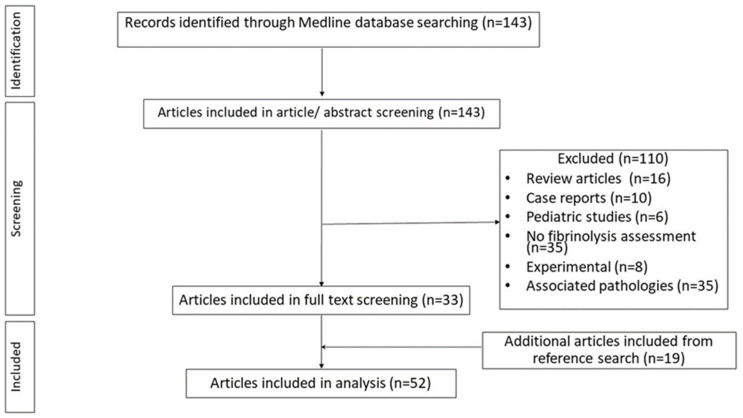
Flowchart of the article selection process.

Search strategy retrieved 143 articles, from which only 33 were included. The remaining 110 articles were excluded for the following reasons: 35 articles did not assess fibrinolysis, 10 were case reports, 16 were reviews, 6 were pediatric studies, 8 were experimental, and 35 described patient populations having associated pathologies possible leading to fibrinolysis alterations (Figure 1). Additional eligible studies were identified by searching the reference lists of articles that were fully assessed. 19 additional articles were added from references, resulting in 52 articles included in our review (Figure 1). Table 1 summarizes the different techniques used to assess fibrinolytic activity in the research studies examined.

The 52 studies included in this review encompassed diverse patient populations and clinical settings. Studies included surgical ICU patients (n = 8 studies), medical ICU patients (n = 15 studies), mixed ICU populations (n = 12 studies), emergency department patients (n = 4 studies), and general hospitalized septic patients (n = 13 studies). Patient populations ranged from 7 to 775 participants, with most studies including patients with sepsis and/or septic shock. Studies were predominantly observational cohorts (n = 45), with 7 randomized controlled trials examining therapeutic interventions such as glycemic control.

The methods used for fibrinolysis assessment in adult patients with bacterial sepsis can be grouped in two main categories: measurement of plasma biomarkers involved in fibrinolysis and tests assessing global fibrinolytic potential, which can be further classified based on the type of blood sample: plasma, euglobulin fraction of plasma, and whole blood. The strengths and limitations of the different methods used for fibrinolysis assessment in adult patients with bacterial sepsis are shown in Table 2.

### 3.1. Measurement of Plasma Biomarkers Reflecting Fibrinolysis

These involve measuring the levels of specific proteins, their precursors, or degradation products in plasma that are part of the fibrinolytic system. These markers provide specific information about various components and stages of the fibrinolytic pathway. Overall concentrations are assessed using antigen-based tests, while various specific functional assessments evaluate their activity [5,93]. These biomarkers can complement global tests by providing insights into the mechanisms behind fibrinolysis disturbances. However, despite the wide range of available biomarker measurements, it remains challenging to determine the contribution of individual fibrinolytic factors to the overall fibrinolytic outcome due to the complex and dynamic interactions among different factors involved in the clotting and lysis processes [93]. Additionally, the levels of fibrinolytic biomarkers are influenced by their clearance rates, and interpreting these values becomes even more complicated in conditions characterized by increased coagulation activation, such as sepsis. Since most fibrinolytic biomarkers are assessed from plasma, these tests do not account for the intricate interactions between plasma and cellular factors that contribute to fibrinolysis. Furthermore, there are concerns regarding the lack of standardization for some tests, such as D-dimer testing, as well as the limited availability of certain plasma biomarker tests, such as PAI-1. The measurement methods and clinical usefulness of plasma biomarkers reflecting fibrinolysis are summarized in Table 3.

#### 3.1.1. Fibrin Degradation Products in Sepsis

The fibrinolysis-related biomarkers, such as D-dimers and fibrin degradation products, are dependent on both fibrinolytic activity and coagulation activation, as well as their clearance from the circulation [88]. This may explain the elevated markers of fibrinolytic activity observed in septic patients [76,79,86,94]. Semeraro et al. studied 271 sepsis patients categorized by D-dimer levels: no increase (<500 ng/mL, n = 7), moderate increase (500–4000 ng/mL, n = 122), marked increase (>4000 ng/mL, n = 142) [88]. Their results indicated that septic patients with normal D-dimer levels exhibited stronger fibrinolysis inhibition, as evidenced by lower levels of PAP and higher PAI-1 compared to those in the other D-dimer groups [88]. Furthermore, patients with normal D-dimer levels had higher mortality rates than the other groups, suggesting that severe fibrinolysis inhibition, which prevents the rise in D-dimer levels, correlates with organ dysfunction and poorer prognosis [88]. In another study, Semeraro et al. measured various clotting and lysis biomarkers, including PAP, D-dimer (DD), and prothrombin fragment 1+2 (F1+2) [89]. They employed a novel method to reflect the balance between coagulation and fibrinolysis using a corrected D-dimer formula for thrombin and plasmin formation (DDcorr = DD × PAP/F1+2) [89]. The results revealed highest mortality in patients with the DDcorr values in first tertile (indicating low fibrinolysis), with intermediate mortality in the third tertile (high fibrinolysis) and the lowest mortality in the second tertile (balanced fibrinolysis). This indicates that an imbalance in the coagulation-fibrinolysis system increases mortality risk [89].

#### 3.1.2. Plasminogen Activators and Inhibitors in Sepsis

PAI-1 can be measured using two different approaches: PAI-1 antigen assays measure the total concentration of PAI-1 protein (both active and inactive forms), while PAI-1 activity assays specifically measure the functionally active PAI-1 that can inhibit plasminogen activators [93]. In sepsis, both approaches are valuable, as elevated PAI-1 antigen indicates increased production, while PAI-1 activity directly reflects the functional inhibition of fibrinolysis.

In addition to elevated levels of t-PA and PAP, increased concentrations of PAI-1 have also been observed in patients with sepsis [7,35,66,86,94,95]. Gould et al. further reported that PAI-1 levels were significantly higher in septic patients compared to controls and were positively correlated with elevated concentrations of CFDNA [49]. In a prospective study involving 14 consecutive patients with septic shock, researchers conducted serial measurements of plasma concentrations of PAI-1, PAP, and tPA [59]. Other parameters measured included thrombin-antithrombin (TAT) complexes, and lactate levels. The findings indicated that PAI-1 was elevated in 77% of the measurements, while tPA and PAP were increased in 51% and 48% of the measurements, respectively [59]. The increased activation of coagulation (as indicated by elevated TAT levels) and the inhibition of fibrinolysis (high PAI-1) were found to be predictors of hyperlactatemia in multivariate regression analysis [59]. Furthermore, non-survivors exhibited persistently elevated levels of PAI-1 and lactate [59]. Several studies, including a systematic review and meta-analysis, have shown that septic patients with more severe disease and non-survivors have higher levels of PAI-1 compared to survivors [38,42,62,64,65,67,72,74,75,76,90,94,95,96]. In their study, Schmitt et al. found increased levels of t-PA and PAI-1 in postoperative patients and septic patients compared to healthy volunteers in the first week after surgery and, respectively, after sepsis onset [86]. The increase in PAI-1 was more important in sepsis than in postoperative patients, with the highest plasmatic levels in the first 24 h after sepsis onset [86]. According to Zeerleder et al., PAI-1 is the main inhibitor of the fibrinolytic process in early sepsis, while TAFI might be responsible for ongoing fibrinolysis inhibition in the later stages [42]. Another study assessed total TAFI, activated and inactivated TAFI (TAFIa and TAFI ai) levels in septic patients and controls [80]. This method allows for easy monitoring of changes in TAFI activation, as thrombin/thrombomodulin or plasmin convert TAFI into TAFIa, which then inactivates to TAFIai, accumulating in the activation pathway. The results demonstrated higher levels of TAFIa/ai in plasma samples from patients with sepsis than in samples from healthy controls, suggesting the up-regulation of the TAFI activation pathway in sepsis [80].

While the correlation between low platelet number and mortality is well known in septic patients, Semeraro et al. demonstrated that fibrinolysis impairment preceded the development of thrombocytopenia in septic patients [87]. In their study, septic patients with early or late thrombocytopenia had higher levels of PAI-1, increased TAFI activation and longer clot lysis time compared to septic patients without thrombocytopenia [87]. In this way, the fibrinolytic shutdown could suggest the onset of clinically relevant thrombocytopenia in sepsis, which is an important predictor of mortality [87].

According to Helling et al., reduced plasminogen levels during the initial phases of shock, along with a failure to increase fibrinolysis (demonstrated by PAP levels) through-out treatment, correlated with a fatal outcome in situations involving septic or hemor-rhagic shock [61].

Hyperglycemia in sepsis can influence fibrinolytic activity through multiple mechanisms, including increased PAI-1 synthesis and altered endothelial function, making glycemic control a potential therapeutic target for improving fibrinolytic balance. In a prospective, randomized trial involving 90 patients, Savioli et al. investigated whether tight glycemic control could restore normal fibrinolysis in patients with sepsis [83]. At the time of enrollment, fibrinolysis was inhibited in only a small number of patients (34 out of 90). In the tight glycemic control group, a small but statistically significant improvement in fibrinolysis was observed, evidenced by a decrease in PAI-1 activity and concentration, as well as an increase in PAP complexes [83]. Both groups had similar levels of D-dimer [83]. Another prospective study shows comparable results, indicating that glycemic control may partially reverse fibrinolytic impairment and reduce morbidity in cases of sepsis [82].

#### 3.1.3. Fibrinolysis Biomarkers and Disseminated Intravascular Coagulation (DIC)

In a large cohort of patients with DIC, both septic and non-septic, levels of fibrinogen, fibrin degradation products, and D-dimer were significantly lower, while PAI-1 levels were significantly higher in cases of sepsis-induced DIC than in non-septic DIC cases [73]. Additional research has indicated that impaired fibrinolysis, specifically through elevated PAI-1 levels, is associated with overt DIC [38,63]. Mei et al. included in their study 444 patients with suspected DIC, of which 77 were septic [77]. From the sepsis patient subgroup, 32 had overt DIC, and 42 had non-overt DIC. The levels of tPA-PAI-1 complexes and TAT complexes were significantly higher in patients with overt DIC compared to those with non-overt DIC, while the levels of PAP complexes were similar. These findings indicate impaired fibrinolysis due to increased PAI-1 activity, resulting in microthrombus formation and organ failure [77]. The lack of difference in the levels of PAP complexes suggests that coagulation is highly activated (as evidenced by elevated TAT) in overt DIC. At the same time, effective plasmin generation is suppressed, contributing to poor outcomes [77].

In a study involving 50 patients with sepsis or septic shock and healthy controls, TAFI activity and antigen, D-dimer, the neutrophil elastase-alpha1-proteinase inhibitor complex (neutrophil elastase), fibrin degradation product by neutrophil elastase (E-XDP), PAP, and the t-PA-PAI-1 complex were measured [60]. The results showed lower TAFI activity and higher levels of soluble fibrin, neutrophil elastase, E-XDP, PAP, the t-PA-PAI-1 complex, and D-dimer in DIC compared to non-DIC patients [60]. Low TAFI activity and high neutrophil elastase independently predicted death or organ dysfunction, linking impaired fibrinolysis to poor outcomes [60]. Another study revealed that E-XDP levels correlate with the prognosis in sepsis-induced DIC, underlining the contribution of leukocyte elastase to the degradation of cross-linked fibrin in sepsis [44]. Gando et al. included in their study 45 consecutive patients with systemic inflammatory response syndrome or sepsis admitted to the ICU, of which 11 had DIC [47]. Fibrin degradation products by neutrophil elastase (E-XDP) and by plasmin (FDP), D-dimer, soluble fibrin, and PAI-1 plasma levels were assessed [47]. DIC patients showed significantly higher levels of peak E-XDP, FDP, soluble fibrin, and PAI-1 compared to non-DIC patients. These findings suggest that the activation of fibrinolysis (by both plasmin and neutrophil elastase) was insufficient to overcome the PAI-1-mediated fibrinolytic shutdown in DIC patients [47]. Another study included patients with septic shock and DIC and healthy controls, and assessed human neutrophil elastase (HNE)-DNA complexes (from NETs), plasminogen, plasminogen fragments, and HNE-α1-proteinase inhibitor (HNE-α1-PI) levels [56]. Patients experiencing septic shock and DIC have been observed to have circulating HNE-DNA complexes, as well as HNE-derived plasminogen fragments. These patients also exhibit low levels of plasminogen and a diminished ability to generate plasmin on fibrin when compared to healthy controls. This evidence highlights the significant role of NETs in lowering plasminogen levels and impairing fibrinolysis during severe infections [56].

#### 3.1.4. Fibrinolysis Biomarkers in Melioidosis

Melioidosis commonly affects patients with diabetes, and diabetes itself can influence hemostatic function, making it essential to understand whether pre-existing diabetes affects coagulation and fibrinolysis in septic patients. Studies on patients with melioidosis (Burkholderia pseudomallei infection), a common cause of community-acquired sepsis in Southeast Asia and northern Australia, demonstrated abnormalities in fibrinolysis (increased D-dimer and PAP) with activation of coagulation (elevated TAT, prothrombin fragment F1+2 and fibrinogen levels) compared to healthy controls [70]. Consistent with prior research, D-dimer and PAP levels were also higher in non-septic diabetics compared to healthy controls [70]. However, pre-existing diabetes did not influence the hemostatic alterations in the context of septic melioidosis [70]. Similar results were observed in another study, which found that concentrations of tPA, PAI-1, D-dimer, and PAP were significantly elevated in patients with melioidosis compared to healthy controls [92]. The authors emphasized that the tPA assay used also detects tPA that is complexed with its inhibitor, PAI-1, which contributes to the high levels of tPA antigen found. They concluded that the substantial release of PAI-1 likely leads to a net suppression of fibrinolysis in cases of melioidosis [92]. Similarly to other causes of sepsis, melioidosis patients who exhibited a predominance of the prothrombotic pathway (high ratio of TAT to PAP levels) had poorer prognoses [92].

### 3.2. Global Tests of Fibrinolysis in Sepsis

The overall assessment of fibrinolytic activity is best represented by global tests of fibrinolysis, which can be conducted using plasma, the euglobulin fraction of plasma, or whole blood [5,8]. Unlike clotting, fibrinolysis typically occurs at a slow rate. To facilitate its assessment in a reasonable timeframe, fibrinolytic inhibitors are often removed, or activators are added [5]. However, this approach may alter the natural endogenous “in vivo” fibrinolytic process and complicate the execution of fibrinolytic tests. Most global fibrinolytic assays, with the exception of the VET, are technically challenging and time-consuming to perform [5].

#### 3.2.1. Global Tests of Fibrinolysis from Plasma

The measurement of fibrinolytic activity is often based on the changes in optical density over time, reflecting clot formation and lysis, after the simultaneous addition of agonists to initiate clotting (e.g., thrombin, tissue factor, Ca++) and fibrinolysis (e.g., tPA or uPA) to citrated plasma [8,93]. The parameter often reported in research using plasma based fibrinolytic tests is the clot lysis time (CLT), which represents the time from 50% of maximal clotting to 50% lysis [8]. Plasma-based fibrin clot formation and lysis provides a comprehensive insights into both fibrin formation and lysis, it is sensitive to both hyper- and hypofibrinolysis, and can be modified by adding different inhibitory molecules to address various research questions [93]. However, it is primarily a research tool, not being used routinely in clinical practice due to lack of automation, and standardization, and also long turnaround times.

A plasma-based clot formation and lysis assay was used by Larsen et al. to assess fibrin formation and lysis in 34 septic shock patients [71]. Three distinct clot–lysis profiles were described in their patient group: severely decreased fibrin formation (flat curve) in 30% of the patients, normal curve in 38% of cases, and pronounced lysis resistance (lysis-resistant curve) in 32% of patients [71]. This last group had normal or increased fibrin formation with fibrinolysis resistance, lower plasma plasminogen and higher PAI-1 levels than patients with normal curves [71]. In their study, Semeraro et al. found increased CLT in septic patients with thrombocytopenia, who also had higher plasma concentrations of PAI-1 and a higher mortality rate compared to non-thrombocytopenic septic patients [87]. Increased CLT, as assessed by the turbidimetric method, was also observed in septic patients in a study by Gould et al., where the plasmatic concentration of CFDNA correlated with the decrease in plasmin-mediated fibrin lysis [49].

Similarly to thrombin generation tests, plasmin generation tests allow for the direct measurement of plasmin formation and inhibition kinetics in plasma [93,97]. In these tests, coagulation and fibrinolysis are initiated by the addition of exogenous TF and tPA, respectively [93]. As in thrombin generation, plasmin generation is assessed by the cleavage of a fluorogenic substrate [93]. There are various tests available, some of which detect both thrombin and plasmin simultaneously. In a recent study, Bouck et al. performed a comprehensive hemostatic evaluation of 46 COVID-19 patients, 53 sepsis patients, and 18 healthy volunteers [52]. Fibrinolysis was evaluated using D-dimer, PAP complexes, as well as plasma-based tests for plasmin generation and turbidimetric clot lysis [52]. Both patient groups exhibited high levels of plasmatic markers indicating fibrinolytic activity; however, their dynamics varied. Plasma from COVID-19 patients generated thrombin and plasmin more rapidly, while plasma from sepsis patients demonstrated delayed plasmin generation and longer fibrin-lysis lag times [52].

Using a modified ROTEM test with added t-PA in platelet-poor plasma samples, Panigada et al. demonstrated impaired clot lysis in septic patients compared to controls, showing that impaired dynamics of clot formation and hypofibrinolysis coexist and are strictly related in severe sepsis [36].

#### 3.2.2. Global Tests of Fibrinolysis from the Euglobulin Fraction of Plasma

Euglobulin clot lysis time (ECLT) is a test commonly used for assessing fibrinolysis. It is performed using the euglobulin fraction of plasma obtained from platelet-poor plasma through dilution, acidification, and centrifugation processes. This results in a product with relatively preserved levels of plasminogen activators and reduced amounts of PAI-1 [8]. This method allows fibrinolytic activity assessment without the addition of exogenous fibrinolytic activators; however, obtaining the euglobulin fraction of plasma is time-consuming, and the test is only conducted in specialized laboratories. The ECLT is used to evaluate endogenous tPA activity, which is affected by the balance between plasma levels of tPA and PAI-1 [93]. Low fibrinolytic activation revealed by prolonged ECLT was observed in septic patients compared to healthy controls or to non-septic critically ill patients [35,53].

#### 3.2.3. Global Tests of Fibrinolysis from Whole Blood

The main components of the fibrinolytic system are found in plasma; however, whole blood tests offer a significant advantage by providing a more comprehensive view. They include the important contributions of circulating cells, particularly platelets, which release PAI-1 and TAFI, and can modify clot structure through platelet-mediated clot retraction. This holistic approach is invaluable for understanding the fibrinolytic process [8,98]. For the assessment of fibrinolytic activity in whole blood, studies involving septic adult patients have employed viscoelastic tests (VET) and rheometry [8,97].

VET are very convenient and rapid methods to assess fibrinolysis. Whole-blood VET are closer to the “in vivo” conditions compared to the tests performed from plasma, as they also reflect the contribution of cells; however, they fail to reflect the important contribution of other elements found “in vivo” such as the blood flow or the endothelium. The most well-known VET are thrombelastography (TEG) and rotational thromboelastometry (ROTEM). Although there are differences in the parameters reporting fibrinolysis in TEG and in ROTEM, they are based on the same concept: fibrinolysis is estimated by clot lysis indices reflecting the decrease in clot amplitude related to the maximum amplitude reached during measurement [8].

Studies performed in septic patients typically report lower clot lysis, as assessed by ROTEM or TEG, compared to healthy controls or non-septic patients [7,37,50,54,84,86,91,99]. When comparing the fibrinolytic activity assessed by VET between septic patients with and without DIC, the studies report heterogeneous results. In some studies, the lysis indices assessed by VET were not different between septic patients with and without DIC, both categories having low clot lysis [68,78,85,91]. In a recent study, Koami et al. reported that hypofibrinolysis (defined as ROTEM LI60 ≥ 97%) was strongly associated with positive ISTH (International Society on Thrombosis and Haemostasis) overt DIC, higher SOFA scores and 28-day mortality compared to patients with normal (LI60 86–96%) or increased fibrinolysis (LI60 ≤ 85%) [69]. Schmitt et al. reported that septic patients with overt DIC exhibited lower levels of fibrinolytic activation, as assessed by ROTEM clot lysis indices, compared to septic patients without overt DIC. Furthermore, a more pronounced fibrinolytic shutdown was observed in septic shock patients when compared to both postoperative patients and healthy controls [86]. The fibrinolytic shutdown was also more prevalent among patients with greater disease severity, specifically those with a SOFA score of 18 or higher, compared to patients with lower SOFA scores [86]. Similar results were obtained by Davies et al. in a study on septic critically ill patients, showing impaired fibrinolysis assessed by clot lysis at 60 min using ROTEM in septic shock patients compared to less severe septic patients and in non-survivors compared to survivors [58]. Scarlatescu et al. observed that patients with sepsis exhibited decreased clot lysis compared to control subjects. They developed a new early kinetic parameter, known as t-AUCi, which measures the time required to reach maximal clot amplitude after achieving the highest clot formation velocity [84]. This new parameter showed higher values in sepsis patients than in controls, indicating hypofibrinolysis, and it was a better predictor of ICU mortality than clot lysis indices and the SOFA score [84].

In a group of septic patients, Prakash et al. demonstrated inhibition of fibrinolysis across increasing severity of organ failure, both at presentation and prospectively, indicated by decreasing ML on non-activated ROTEM test (NATEM) and increasing levels of functionally active PAI-1 (aPAI-1) [81]. Furthermore, improvement in sepsis-related organ failure was strongly associated with an early increase in fibrinolysis (increase in ML and decrease in aPAI-1). The findings suggest that monitoring fibrinolysis using a point-of-care device, such as ROTEM, could help identify patients with severe sepsis who may benefit from fibrinolysis augmentation [81]. Adamzik et al. demonstrated lower clot lysis reflected by lysis index at 60 min (LI60) on ROTEM in septic patients compared to postoperative patients and healthy controls; in the same study, clot lysis was similar in healthy controls and postoperative patients and the lysis index was useful to discriminate septic patients from postoperative patients and probands with a better accuracy compared to the conventional biomarkers procalcitonin, interleukin 6, and C-reactive protein [50]. Brenner et al. obtained similar results in their study showing lower fibrinolysis in septic patients than in postoperative patients and better ability of ROTEM clot lysis indices both at 45 and at 60 min to differentiate between sepsis and postsurgical inflammatory response compared to procalcitonin and C-reactive protein [54]. In their cohort of patients with sepsis or septic shock, Andersen et al. demonstrated an overall normocoagulation with ROTEM while the conventional coagulation tests and thrombin generation tests reflected hypocoagulation [51]. In this study, the fibrinolysis was assessed by LI60 in ROTEM, and by D-dimer measurements [51]. The clot lysis index assessed increased from Day 1 to Day 3, suggesting low fibrinolytic activity, while the levels of D-dimers did not show significant changes between measurements [51].

The sensitivity of VET for detecting hyperfibrinolysis was questioned after Raza et al. found that hyperfibrinolysis diagnosed with ROTEM only occurred in trauma patients with significantly elevated PAP complexes, reaching 30 times the normal value [100]. While the ability of VET to detect increased fibrinolytic activity is still under discussion, diagnosing low fibrinolytic activation using VET may prove to be even more challenging. This difficulty arises because normal, healthy individuals typically show minimal clot lysis at baseline, and changes in clot amplitude during measurement are subtle. In a study reporting ROTEM data in healthy individuals from different centers, Lang et al. found the clot lysis index values at 30 min between 95 and 100% and maximum lysis values from 0 to 18% [6]. VET provides a global assessment of hemostasis by recording clot amplitude over time. The measurement of fibrinolysis at a specific moment is related to the clot amplitude at that moment compared to the maximum amplitude achieved. This reflects the balance between clot formation and degradation at that particular time. Therefore, if clot formation is ongoing, the existing fibrinolytic activity may be obscured, resulting in clot lysis values that fall within the normal or fibrinolysis shutdown range.

In several studies, modifying viscoelastic testing (VET) by incorporating fibrinolysis activators has been employed to enhance the assessment of fibrinolysis, improving the detection of fibrinolysis resistance [7,35,36,37,55,101]. Until now, only one standardized assay using viscoelastic technology and tests incorporating tPA is commercially available and approved for diagnostic use [101]. In their study, Kuiper et al. performed tissue factor-activated ROTEM in healthy volunteers and in different clinical categories of patients, including septic patients [35]. When the test was performed without adding fibrinolysis activators, no differences regarding the fibrinolytic potential were seen in septic patients compared to healthy controls within a runtime of 2 h [35]. On the contrary, when ROTEM was spiked with recombinant t-PA, clot lysis time was prolonged in sepsis compared to healthy controls, revealing fibrinolytic resistance [35]. Similar findings were reported by Scarlatescu et al. in a study comparing standard and modified ROTEM tests in sepsis patients and healthy controls [7]. Brewer et al. used ROTEM with added tPA to investigate fibrinolysis in 159 intensive care (ICU) patients, of which 30 had sepsis [55]. Parameters measured included lysis time (LT), maximum lysis (ML), and fibrinolysis speed (FS), and low fibrinolytic activity was defined as an LT above the 97.5th percentile of healthy individuals (50 min) [55]. Their results showed impaired fibrinolysis in sepsis patients compared with non-sepsis patients, and in ICU patients in general compared to healthy controls. Additionally, impaired fibrinolysis was associated with higher 30-day mortality and venous thromboembolism risk [55].

In another study using non-modified kaolin-activated TEG, lower fibrinolysis was found in sepsis patients compared to healthy individuals, but after adding urokinase to the TEG to stimulate fibrinolysis, the resulting clot lysis indices were variable within the sepsis population, ranging from near normal response to severe fibrinolytic resistance [36]. Therefore, the septic patients were divided into normal and low responders based on the clot lysis produced on TEG after adding urokinase [37]. Low responders exhibited more severe organ dysfunction (SOFA score) and higher mortality [37]. Septic patients demonstrate lower clot lysis when assessed by non-modified VETs. However this is not always correlated with a defective fibrinolytic response to fibrinolytic activators. Identifying septic patients who exhibit fibrinolytic resistance is challenging without utilizing modified VET. This difficulty arises because both categories of septic patients—normal responders and low responders—show similar results when evaluated using standard plasma markers of fibrinolytic activity or non-modified VET [37].

Rheometry is a whole-blood-based method for assessing and quantifying the quality of clot microstructure through its fractal dimension (df) [102]. In a study involving 100 patients with sepsis and septic shock, as well as 44 healthy controls, the fractal dimension (df) was significantly higher in sepsis, indicating increased mechanical clot strength and elasticity consistent with more resistant clots compared to those in septic shock patients [57]. Conversely, df was significantly lower in septic shock and correlated with higher 28-day mortality [57]

## 4. Conclusions and Future Directions

Fibrinolytic dysfunction is a critical but often underdiagnosed aspect of sepsis-induced coagulopathy, significantly affecting patient outcomes. This comprehensive review of current assessment methods highlights the importance of evaluating fibrinolytic activity while also addressing the considerable challenges that hinder its routine use in managing sepsis. Evidence clearly shows that fibrinolytic shutdown, primarily caused by the overproduction of PAI-1, occurs early in the progression of sepsis. This shutdown acts as both a pathophysiological driver of microthrombosis and a valuable prognostic marker for assessing disease severity and mortality. Exaggerated clotting and fibrinolysis impairment are equally important in the pathophysiology of sepsis-induced organ failure. However, the methods for fibrinolysis assessment are less developed than coagulation tests, leaving the evaluation of fibrinolytic activity in sepsis patients in a blind spot.

An important consideration in fibrinolytic assessment during sepsis is the potential contribution of pathogen-derived fibrinolytic factors. Certain bacteria produce their own fibrinolytic enzymes (such as streptokinase from Streptococcus species). These pathogen-associated fibrinolytic factors can theoretically confound measurements of host fibrinolytic activity and may explain some of the heterogeneity observed in fibrinolytic responses among septic patients with different causative organisms. However, in clinical practice, the predominant finding in bacterial sepsis remains fibrinolytic shutdown mediated by host-derived PAI-1, suggesting that pathogen-associated fibrinolysis is generally insufficient to overcome the robust host fibrinolytic inhibition. Future studies should consider pathogen-specific analysis to better understand these complex interactions and their impact on fibrinolytic assessment.

Currently, there are various methods for assessing fibrinolysis, each with distinct advantages and limitations. While plasma biomarker measurements offer valuable mechanistic insights, they are hindered by issues related to standardization, limited availability, and their inability to accurately reflect the final image on the dynamic balance of clotting and fibrinolysis. Global plasma-based tests provide a thorough evaluation of fibrinolytic capacity but are technically complex and not suitable for point-of-care use. In contrast, whole-blood viscoelastic testing, especially when enhanced with fibrinolytic activators, appears to be the most viable option for clinical application. This method enables real-time assessment and can be seamlessly integrated with existing coagulation monitoring systems. The research on this topic is progressing with older techniques such as VET being adapted to become more sensitive for fibrinolysis detection, but also with the development of new techniques such as simultaneous thrombin and plasmin generation assays or whole-blood global fibrinolytic capacity. The integration of fibrinolytic assessment with other hemostatic parameters through comprehensive coagulation monitoring platforms represents another promising direction. Combined thrombin and plasmin generation assays, coupled with viscoelastic testing and biomarker panels, could provide unprecedented insights into the dynamic balance between coagulation and fibrinolysis throughout sepsis progression.

Future research priorities should focus on several critical areas. First, the development and validation of standardized, automated assays suitable for routine clinical practice remains paramount. This includes establishing reference ranges, optimizing testing protocols, and ensuring inter-laboratory reproducibility. Standardization efforts must address the current fragmentation in fibrinolytic testing methodologies. International consensus on testing protocols, reference standards, and clinical decision algorithms would facilitate widespread adoption and enable meaningful comparison of research findings across institutions. Second, large-scale clinical trials are needed to evaluate the impact of fibrinolytic-guided therapeutic interventions on patient outcomes. The emergence of precision medicine approaches offers exciting possibilities for personalized sepsis management. From a clinical perspective, identifying fibrinolytic inhibition through the various assessment methods discussed and real-time fibrinolytic monitoring could guide several therapeutic approaches, such as targeted fibrinolytic enhancement in patients with documented fibrinolytic shutdown (by plasminogen activation or PAI-1 inhibition), supportive measures (improved glycemic control to reduce PAI-1 production), or anticoagulation optimization based on fibrinolytic status.

In conclusion, while significant challenges persist in the methodology of fibrinolytic assessment and its clinical implementation, the evidence strongly supports the clinical importance of evaluating fibrinolysis in sepsis. The ongoing development of testing technologies, standardization efforts, and therapeutic innovations positions fibrinolytic assessment as a key component of future precision medicine approaches for managing sepsis. Successfully integrating these advances into routine clinical practice will require sustained collaboration among researchers, clinicians, industry partners, and regulatory bodies. The ability to assess fibrinolytic activity can guide personalized treatments, predict clinical outcomes, and enhance sepsis management, presenting a significant opportunity to positively influence patient care.

## Figures and Tables

**Table 1:** Methods of fibrinolysis assessment in sepsis.

Article	Study Population	Fibrinolysis Assessment Method	Key Findings	Relationship with Outcomes
Adamzik 2010 [50]	56 sepsis ICU pts, 52 PO controls	ROTEM NATEM +heparinase: LI60	LI60 increased in sepsis compared to PO controls	LI60 > 96.5% predicted sepsis; early diagnostic value
Andersen 2014 [51]	36 ICU sepsis or septic shock pts; no controls	ROTEM LI45. D-dimer on days 1, 2, 3, 7	LI45 increased from Day 1 to Day 3; D-dimers had increased values (>7 × ULN)	Pts with overt DIC had lower LI45 than pts without overt DIC
Bouck 2021 [52]	46 COVID-19 pts, 53 sepsis pts (52 with bacterial sepsis), 18 healthy controls	Plasma PG, D-dimer, PAP, plasma-based turbidimetric clot-lysis	Both cohorts: increased D-dimer and PAP; in sepsis pts delayed and decreased PG	Longer fibrinolysis lag linked to higher SOFA and longer ICU LOS
Boudjeltia 2004 [53]	11 septic ICU pts, 21 non-septic ICU pts;	ECLT	ECLT prolonged in sepsis compared to non-septic pts;	ECLT positively correlated with CRP and ICU LOS
Brenner 2012 [54]	30 septic shock pts; 30 surgical pts; 30 healthy volunteers	ROTEM LI45, LI60;	Increased LI45, LI60 in sepsis;	LI45 predicted sepsis diagnosis
Brewer 2024 [55]	159 ICU pts (30 sepsis, 129 non-sepsis), 38 healthy controls	ROTEM modified with exogenous tPA (tPA-LT, FS)	61% of ICU pts had prolonged LT > 50 min; significantly impaired fibrinolysis activity in sepsis pts compared to ICU pts without sepsis and controls	LT > 97.5th percentile associated with 30-day mortality and VTE
da Cruz 2019 [56]	7 septic shock pts with DIC, 10 healthy controls	NET-bound elastase assay; plasminogen; plasminogen fragments; PG onto fibrin	Pts with DIC had HNE-DNA complexes, HNE-derived plasminogen fragments, lower plasminogen concentration, and capacity to generate plasmin onto fibrin compared to controls	Presence of plasminogen fragments and reduced PG linked to DIC severity
Davies 2016 [57]	100 pts (70 sepsis, 30 septic shock), 44 healthy controls	Fractal dimension (df) rheometry	Sepsis: increased df (dense hypercoagulable clots, resistant to lysis); septic shock: decreased df (weak clots)	Lower df associated with 28-day mortality
Davies 2018 [58]	100 pts (70 sepsis, 30 septic shock)	ROTEM EXTEM/INTEM (CT, MCF, LI60)	Sepsis: increased MCF (hypercoagulable); septic shock: prolonged CT & impaired lysis (LI60)	LI60 > 95% and prolonged CT associated with 28-day mortality
Gando 2007 [47]	45 SIRS/sepsis patients (11 DIC, 34 non-DIC)	E-XDP, FDP, D-dimer, PAI-1, soluble fibrin	Increased soluble fibrin, PAI-1, FDP, Peak E-XDP in DIC compared to non-DIC pts	DIC correlated with organ dysfunction/mortality. Peak E-XDP inversely correlated with death
Gould 2015 [49]	60 sepsis pts; 10 healthy controls	plasma-based turbidimetric clot-lysis; D-dimer, PAI-1;	CFDNA >5 µg/mL impaired fibrinolysis (dense clots; reversible with DNase). Plasmin-CFDNA-fibrin ternary complex inhibited lysis.	NA
Hartemink 2010 [59]	14 septic shock pts	tPA, PAI-1, PAP, fibrinogen	PAI-1 time course predicted lactate independent of hemodynamics/inflammation.	High PAI-1persisted in non-survivors
Hayakawa 2012 [60]	50 sepsis pts (37 DIC, 13 non-DIC); 15 healthy controls	PAI-1, TAFI antigen and activity, E-XDP, PAP, tPAIC, D-dimer	decreased TAFI activity and insufficient fibrinolysis activation by plasmin/neutrophil elastase; increased PAI-1, E-XDP, tPAIC, D-dimer in DIC	High neutrophil elastase and low TAFI, PAP independently predict death and OD; tPAIC predicts E-XDP elevation
Helling 2010 [61]	39 pts (21 hemorrhagic shock, 18 septic shock)	Plasminogen, PAP, D-dimer	Decreased plasminogen in non-survivors (day 1); plasminogen levels, PAP continuously increasing in survivors up to day 7	Plasminogen recovery correlates with survival;
Hesselvik 1989 [62]	53 sepsis pts	PAI-1, α2-plasmin inhibitor, D-dimer.	Plasminogen levels lower, and PAI-1 levels higher in septic shock compared to sepsis pts	High PAI-1 correlates with mortality
Hoppensteadt 2013 [63]	617 sepsis pts (98 overt DIC, 518 non-overt DIC); 30 healthy controls	PAI-1	Increased PAI-1 in overt DIC;	High PAI-1 correlates with overt DIC
Hoshino 2017 [64]	186 sepsis pts (150 survivors, 36 non-survivors)	PAI-1, D-dimer, PAP	PAI-1 increased in non-survivors	PAI-1 ≥83 ng/mL independently predicted 28-day mortality in sepsis
Hoshino 2020 [65]	113 sepsis pts (61 PAI-1 ≥83 ng/mL, 52 PAI-1 <83 ng/mL)	PAI-1, D-dimer, PAP	PAI-1 ≥83 ng/mL linked to pre-DIC state, OD	Increased PAI 1 correlated with lower ICU/CRRT/catecholamine-free days and with decreased 28-day survival
Iba 2005 [66]	78 surgical ICU pts with sepsis (15 with OD, 63 without);	D-dimer, FDP, PAP, total PAI-1, E-XDP	Patients with OD had significantly higher D-dimer, FDP, PAP, PAI-1, E-XDP.	Early elevation of D-dimer, FDP, E-XDP, and PAI-1 associated with OD and mortality.
Johansson 2014 [67]	67 septic pts (14 with, 53 without noradrenaline infusion)	tPA, PAI-1	Endogenous noradrenaline correlated with increasedPAI-1, tPA, and endothelial damage markers.	High PAI-1 associated with higher 28- and 90-day mortality.
Koami 2015 [68]	13 septic pts (7 DIC, 6 non-DIC)	ROTEM EXTEM LI30, 45, 60, ML; FDP; D-dimer	Higher D-dimer and FDP in DIC than in non-DIC	LI30, 45, 60, ML were not significantly different between DIC and non-DIC
Koami 2025 [69]	63 septic ICU pts (lysis classified by EXTEM LI60: Hyper ≤85%, Normal 86–96%, Hypo ≥97%)	ROTEM EXTEM LI60; FDP, D-dimer	Hypofibrinolysis pts had higher FDP, D-dimer levels than normofibrinolysis pts + higher APACHEII/SOFA/DIC scores.	LI60 ≥97% predicted DIC and higher 28-day mortality
Koh 2011 [70]	44 pts with culture-proven melioidosis (34 with diabetes, 10 without); 30 healthy blood donors; 52 otherwise healthy diabetes patients	D-dimer, PAP	Higher PAP and D-dimer in meloidosis and in diabetes pts compared to healthy controls	Severity of coagulopathy and fibrinolysis impairment correlated with mortality in melioidosis, but diabetes status did not influence the degree of coagulation/fibrinolysis disturbance or outcomes in septic patients.
Koyama 2014 [38]	77 sepsis pts (37 developed overt DIC within 5 days)	PAI-1, plasminogen, α2-plasmin inhibitor, PAP	Plasminogen, α2-plasmin inhibitor were lower and PAI-1 higher in DIC compare to non-DIC	PAI-1 >269 ng/mL predicted overt DIC and 28-day mortality
Kuiper 2016 [35]	40 healthy volunteers, 21 sepsis, 20 CTS (19 with tranexamic acid), 15 cirrhosis, 7 pregnant pts	ROTEM modified with exogenous tPA (LOT, LT, FS);PAI-1, tPA activity, D-dimers, TAFI; ECLT	Hypofibrinolysis in sepsis (increased ROTEM LOT/LT; high PAI-1 and D-dimers, low tPA activity, prolonged ECLT)	NA
Larsen 2021 [71]	34 septic shock pts	Plasma-based clot-lysis assay; tPA, PAI-1, TAFI	Three distinct profiles: (1) Severely decreased fibrin formation (flat curve), (2) Normal fibrin formation/lysis, (3) Pronounced lysis resistance. Lower plasma levels of plasminogen and higher PAI-1 levels in (3) compared to (2)	Abnormal profiles (flat/lysis-resistant) associated with OD
Lorente 1993 [72]	48 septic shock pts (25 nonsurvivors)	t-PA, u-PA, PAI-1, plasminogen, α_2_-antiplasmin, D-dimer	Increased t-PA, PAI-1, and low plasminogen, plasminogen/α_2_-antiplasmin ratio	Survivors had progressive normalization of fibrinolysis markers.
Madoiwa 2006 [73]	117 sepsis-induced DIC; 1627 non-septic DIC	Plasma PAI-1, D-dimer, FDP	Increased PAI-1 and decreased FDP, D-dimer in septic DIC vs. non-septic DIC; D-dimer negatively correlated with PAI-1	PAI-1 >90 ng/mL linked to increased 28-day mortality
Madoiwa 2011 [44]	117 sepsis-induced DIC pts; 46 healthy controls	E-XDP, p-XDP; PAI-1, D-dimer, PAP	E-XDP higher in DIC compared to controls; E-XDP not correlated with PAI-1, PAP	e-XDP <3 U/mL correlated with increased 28-day mortality
Massignon 1994 [74]	34 pts (10 PO, 12 sepsis, 12 septic shock); 21 controls	PAI-1 Ag and activity, t-PA activity, FDP, D-dimer, a2- antiplasmin	tPA activity decreased, PAI-1 activity and PAI-1 Ag increased in septic shock patients compared to the other groups	PAI-1 levels correlated with TNF, IL-6 levels and OD
Mauri 2010 [75]	90 ICU pts (sepsis/septic shock)	PTX3, PAI-1	PTX3 higher in septic shock patients compared to sepsis and correlated with PAI-1 and F1+2, indicating coagulation/fibrinolysis dysfunction.	Persisting high PTX3 predicted mortality
Mavrommatis 2001 [76]	82 septic pts (including 17 pts with septic shock), 14 healthy controls	PAP, tPAIC, plasminogen, tPA, PAI-1, D-dimer, FDP, a2- antiplasmin	Compared to sepsis, in septic shock pts plasminogen and tPA were lower, and PAI-1 increased; compared to controls PAI-1, D-dimer, FDP were higher in sepsis	Impaired fibrinolysis correlated with increased sepsis severity
Mei 2019 [77]	74 pts with sepsis (32 overt-DIC and pre-DIC, 42 non-overt DIC); 137 healthy controls	PAP, tPAIC	tPAIC higher in patients with overt and pre-DIC compared to non-overt DIC; no differences in PAP	Higher complexes tPA/PAI-1 levels correlated with higher 28-day mortality
Müller 2019 [78]	23 critically ill pts (13 DIC, 10 non-DIC)	ROTEM (EXTEM, INTEM ML, PAP, D-dimer	No significant differences in ML, PAP, D-dimer between DIC and non-DIC pts	NA
Panigada 2015 [37]	40 sepsis pts (sepsis/septic shock); 40 healthy controls	Kaolin-TEG modified with urokinase (Ly30), PAI-1, TAFI, D-dimer	Fibrinolytic resistance (decreased urokinase-TEG Ly30) in 45% of pts, associated with increased PAI-1 and cellular damage	Low urokinase-TEG Ly30 predicted mortality and higher SOFA scores.
Panigada 2016 [36]	40 severe sepsis/septic shock pts; 50 healthy controls	ROTEM EXTEM from PPP modified with added tPA: LT; D-dimer, PAI-1, plasminogen	prolonged LT, higher D-dimer, PAI-1, lower plasminogen in septic pts vs. controls	No direct survival difference after multiple comparisons correction.
Park 1999 [79]	32 sepsis pts, 20 controls	PAP	Increased TAT and PAP in sepsis compared to controls	Higher TAT/PAP ratio in non-survivors than in survivors
Park 2010 [80]	25 sepsis pts; 18 healthy controls	TAFIa, TAFIai, D-dimer, FDP	increased TAFIa/ai in sepsis, no difference in total TAFI compared to controls	NA
Prakash 2015 [81]	77 ICU pts; 20 healthy controls	ROTEM NATEM ML, aPAI-1, D-dimer	aPAI-1 levels higher in sepsis than controls Hypofibrinolysis (low ML) correlated with higher SOFA scores	Lower ML and higher aPAI-1 predicted OD and mortality. Early increase in fibrinolysis correlated with organ recovery.
Sanches 2014 [82]	41 septic shock (21 hyperglycemic, 20 normoglycemic); non-diabetic pts	PAI-1, tPA, plasminogen, α_2_-antiplasmin, D-dimer	Hyperglycemic pts after glycemic control: decreased PAI-1 and tPA, increased plasminogen. Normoglycemic pts: no significant changes in fibrinolysis markers.	Glycemic control associated with improved coagulation/fibrinolysis parameters vs. baseline and vs. normoglycemic patients.
Savioli 2009 [83]	90 ICU pts with sepsis/septic shock (45 tight vs. 45 conventional glycemic control)	PAI-1 activity/concentration, tPA, PAP, D-dimer	34/90 patients had fibrinolysis inhibition (increased PAI-1) at baseline. Tight glycemic control decreased PAI-1 and higher PAP complexes.	Fibrinolysis inhibition linked to increased 90-day mortality
Scarlatescu 2018 [84]	76 septic pts (20 survivors, 56 nonsurvivors); 26 healthy controls	ROTEM EXTEM: LI30, LI45, LI60, ML, t-AUCi	LI30,45,60 higher in sepsis than controls; t-AUCi correlated with clot lysis	t-AUCi predicted ICU mortality, differentiated nonsurvivors vs. survivors even with similar lysis indices
Scarlatescu 2020 [85]	97 septic pts (44 overt DIC, 53 no DIC)	ROTEM EXTEM ML, D-dimer, FDP	FDP and D-dimer higher in DIC than in non-DIC; ML not different between groups	NA
Scarlatescu 2024 [7]	30 septic pts, 30 healthy controls	ROTEM EXTEM, NATEM modified with added tPA, PAI-1, PAP, plasminogen, t-AUCi	Higher PAI-1, PAP, t-AUCi, LI30, 45, 60 and lower plasminogen levels in sepsis compared to controls; t-AUCi >1962 s predicted fibrinolysis resistance.	NA
Schmitt 2019 [86]	90 pts (30 septic shock, 30 surgical controls, 30 healthy volunteers)	ROTEM: LI45, LI60; PAI-1, tPA (total and free)	tPA, PAI-1 increased in surgical controls and septic pts compared to healthy controls; increased LI45/LI60 in sepsis compared to surgical and healthy controls	Increased LI60 predicted mortality
Semeraro 2017 [87]	280 sepsis pts (group 1: baseline thrombocytopenia ≤50 × 10^9^/L group 2: developing thrombocytopenia, group 3: without thrombocytopenia ≥100 × 10^9^/L)	PAI-1, TAFI zymogen, TAFIa/ai, PAP, D-dimer, F1+2, CFDNA, plasma clot lysis time	Fibrinolysis shutdown preceded thrombocytopenia development. Groups 1&2 (thrombocytopenic) vs. Group 3: higher PAI-1 and TAFIa/ai, lower TAFI zymogen, prolonged plasma clot lysis time	TAFI, D-dimer, and PAP were independent predictors of 90-day mortality. Low platelets + low fibrinolysis (low TAFI or PAP) associated with worse outcomes.
Semeraro 2018 [88]	271 sepsis pts grouped by D-dimer levels: no increase (<500 ng/mL, n = 7), moderate increase (500–4000 ng/mL, n = 122), marked increase (>4000 ng/mL, n = 142)	D-dimer, PAI-1, PAP, TAFIa/ai levels	Patients with ‘normal’ D-dimer (<500 ng/mL) had strong fibrinolysis inhibition: lower PAP and higher PAI-1 compared to moderate/marked increase D-dimer groups.	Normal D-dimer group had highest mortality
Semeraro 2020 [89]	269 sepsis pts	DDcorr: Formula DD×PAP/F1+2; D-dimer, PAP	Low DDcorr correlated with low fibrinolytic activity.	Low DDcorr (insufficient fibrinolysis) and high DDcorr (excessive fibrinolysis) associated with higher mortality.
Shaw 2011 [90]	775 sepsis pts stratified by PC deficiency (≤40% vs. >40% activity)	plasminogen, PAI-1, D-dimer	Severe PC deficiency was associated with elevated PAI-1, lower plasminogen, higher D-dimer	Severe PC deficiency and impaired fibrinolysis predicted OD.
Sivula 2009 [91]	28 pts with sepsis (12 with overt DIC), 10 healthy controls	ROTEM EXTEM LI60, D-dimer	LI60 and D-dimer higher in sepsis than controls; Overt DIC pts higher LI60 and D-dimer compared to controls and non-DIC pts	Impaired fibrinolysis associated with overt DIC, higher SOFA and 28-day mortality
Zeerleder 2006 [42]	40 pts (32 sepsis, 8 septic shock); 151 healthy controls	TAFI antigen, PAI-1, PAP, D-dimer;	TAFI antigen was significantly decreased, PAI-1 increased in sepsis vs. controls; PAI-1, PAP higher in septic shock and overt DIC.	High PAI-1 (but not TAFI) and increased TAT/PAP ratio were associated with OD, and mortality.
Wiersinga 2008 [92]	34 pts with culture-proven septic melioidosis; 32 healthy controls	tPA, PAI-1, PAP, D-dimer	Pts had strong activation of coagulation (increased TAT, F1+2), downregulation of anticoagulants (decreased PC, S, AT), and evidence of both activation and inhibition of fibrinolysis (increased tPA, PAI-1, PAP, D-dimer). TAT/PAP ratio higher in pts than controls (prothrombotic state).	Higher tPA, PAP on admission were associated with mortality. High TAT/PAP ratio (procoagulant > fibrinolytic activity) predicted poor outcome.

CT clotting time; df fractal dimension; ECLT euglobulin clot-lysis time; FS fibrinolysis speed; LI45/LI60 lysis index at 45/60 min; LT lysis time; LOT, lysis onset time; MCF maximum clot firmness; NET neutrophil extracellular trap; ROTEM rotational thromboelastometry; TEG, thrombelastography; SOFA Sequential Organ Failure Assessment; tPA tissue plasminogen activator; PG, plasmin generation; CTS, cardio-thoracic surgery; NA, not assessed; E-XDP, Leukocyte elastase-mediated cross-linked fibrin degradation; p-XDP, plasmin-mediated degradation; PO, postoperative; pts, patients; PTX3, pentraxin3; Ly30, lysis at 30 min after maximal amplitude on thromboelastography; PPP, platelet poor plasma; PAI-1, plasminogen activator inhibitor 1; tPAIC, tissue plasminogen activator-plasminogen activator inhibitor-1 complex, PAP, plasmin-α2 plasmin inhibitor complex; TAFI, thrombin-activatable fibrinolysis inhibitor; aPAI-1, functionally active PAI-1; t-AUCi, time from maximal clot formation velocity to zero velocity; CFDNA, cell-free DNA; DDcorr, corrected D-dimer formula; PC, protein C; OD, organ dysfunction; ECLT, euglobulin clot lysis time; HNE, human neutrophil elastase; TAFIa, activated TAFI; TAFIai, inactivated TAFI; TAT, thrombin-antithrombin complex; AT, antithrombin.

**Table 2:** Advantages and disadvantages of the currently used fibrinolytic assessment methods.

Fibrinolysis Assessment Method	Advantages	Disadvantages
Measurement of plasma biomarkers reflecting fibrinolysis	specific information about different components and stages of the fibrinolytic pathway offer mechanistic insights into fibrinolysis disturbances	do not provide dynamic information about the overall fibrinolytic capacity depend on clearance rate plasma-based tests lack of standardization (D-dimers, TAFI) limited availability for some biomarkers
Euglobulin clot lysis time (ECLT)	good for estimating endogenous tPA activity ECLT and Ca++-ECLT used together reflect PAI-1-dependent fibrinolysis inhibition	time consuming limited availability
Plasma-based fibrin clot formation and lysis	simplicity of the reaction system sensitive to hyper and hypofibrinolysis	turn-over time lack of standardization
Plasma-based plasmin generation assay	provides information about the kinetics of plasmin generation specific to plasmin and sensitive to α2AP, fibrin(ogen)	not sensitive to endogenous concentrations of uPA, tPA not sensitive to plasma concentrations of PAI-1 technical complexity lack of standardization limited availability
VET	rapid turnaround time include the contribution of both plasmatic and cellular components	low sensitivity for detecting low fibrinolytic activity
Modified VET (added fibrinolysis activators)	improved capacity for detecting fibrinolytic resistance	lack of standardization increased amount of activators leads to limited detection of minor changes in fibrinolytic homeostasis
Fractal dimension (whole-blood rheometry)	unique information about clot microstructure and mechanical strength	lack of standardization

α2AP, alpha 2 antiplasmin; ECLT, euglobulin clot-lysis time; tPA, tissue plasminogen activator; uPA, urokinaze-type plasminogen activator; PAI-1, plasminogen activator inhibitor 1; TAFI, thrombin-activatable fibrinolysis inhibitor; VET, viscoelastic tests.

**Table 3:** Plasma biomarkers used for fibrinolysis assessment: testing methods and clinical relevance.

Biomarker	Test Method	Current Status in Practice
PAI-1 antigen and activity	ELISA	Reference and research settings, limited clinical use
tPA-PAI-1 complex	ELISA	Reference and research settings, limited clinical use
D-dimer and Fibrin degradation Products	ELISA	Routinely used for DIC/coagulopathy in clinical labs
PAP	ELISA	Reference/research settings, limited clinical use
α2 antiplasmin antigen and functional levels	ELISA (for antigen) Chromogenic methods (for functional levels)	Reference/research settings, not routinely used
TAFI antigen and activity	ELISA	Reference/research settings, limited clinical use Lacks standardization
t-PA antigen and activity	ELISA (for antigen) tPA-specific chromogenic/fluorogenic substrate (for activity)	Reference/research settings, limited clinical use

tPA, tissue plasminogen activator; PAI-1, plasminogen activator inhibitor 1; TAFI, thrombin-activatable fibrinolysis inhibitor; PAP, plasmin-antiplasmin complexes; ELISA, enzyme-linked immunosorbent assay.

## Abbreviations

The following abbreviations are used in this manuscript:

VET	Viscoelastic tests
ROTEM	Rotational thromboelastometry
TEG	Thromboelastography
PAMPs	Pathogen-associated molecular patterns
DAMPs	Damage-associated molecular patterns
TF	Tissue factor
NETs	Netrophil extracellular traps
PAR-1	Protease-activated receptor-1
SIC	Sepsis-induced coagulopathy
DIC	Disseminated intravascular coagulation
tPA	Tissue-type plasminogen activator
uPA	Urokinase-type plasminogen activator
PAI-1	Plasminogen activator inhibitor
TAFI	Thrombin-activatable fibrinolysis inhibitor
CFDNA	Cell-free DNA
TAT	Thrombin-antithrombin complex
PAP	Plasmin-antiplasmin complex
FDP	Fibrin degradation products
ECLT	Euglobulin clot lysis time
LI60	Lysis index at 60 min
LT	Lysis time
ML	Maximum lysis
df	Fractal dimension

## References

[1] Lijnen H.R., Rijken D.C., Rawlings N.D., Salvesen G. (2013). Chapter 646-t-Plasminogen Activator. Handbook of Proteolytic Enzymes.

[2] Chapin J.C., Hajjar K.A. (2015). Fibrinolysis and the control of blood coagulation. Blood Rev..

[3] Lippi G., Favaloro E.J. (2018). Laboratory hemostasis: From biology to the bench. Clin. Chem. Lab. Med..

[4] Williams B., Zou L., Pittet J.F., Chao W. (2024). Sepsis-Induced Coagulopathy: A Comprehensive Narrative Review of Pathophysiology, Clinical Presentation, Diagnosis, and Management Strategies. Anesth. Analg..

[5] Longstaff C. (2018). Measuring fibrinolysis: From research to routine diagnostic assays. J. Thromb. Haemost. JTH.

[6] Lang T., Bauters A., Braun S.L., Potzsch B., von Pape K.W., Kolde H.J., Lakner M. (2005). Multi-centre investigation on reference ranges for ROTEM thromboelastometry. Blood Coagul. Fibrinolysis Int. J. Haemost. Thromb..

[7] Scarlatescu E., Kim P.Y., Marchenko S.P., Tomescu D.R. (2024). Validation of the time to attain maximal clot amplitude after reaching maximal clot formation velocity parameter as a measure of fibrinolysis using rotational thromboelastometry and its application in the assessment of fibrinolytic resistance in septic patients: A prospective observational study: Communication from the ISTH SSC Subcommittee on Fibrinolysis. J. Thromb. Haemost..

[8] Ilich A., Bokarev I., Key N.S. (2017). Global assays of fibrinolysis. Int. J. Lab. Hematol..

[9] Ilich A., Noubouossie D.F., Henderson M., Ellsworth P., Betbadal K.F., Campello E. (2020). Development and application of global assays of hyper- and hypofibrinolysis. Res. Pr. Thromb. Haemost..

[10] Schmoch T., Möhnle P., Weigand M.A., Briegel J., Bauer M., Bloos F., Meybohm P., Keh D., Löffler M., Elke G. (2023). The prevalence of sepsis-induced coagulopathy in patients with sepsis-a secondary analysis of two German multicenter randomized controlled trials. Ann. Intensiv. Care.

[11] Levi M. (2010). The coagulant response in sepsis and inflammation. Hamostaseologie.

[12] Levi M., van der Poll T. (2017). Coagulation and sepsis. Thromb. Res..

[13] Pawlinski R., Mackman N. (2010). Cellular sources of tissue factor in endotoxemia and sepsis. Thromb. Res..

[14] Allen K.S., Sawheny E., Kinasewitz G.T. (2015). Anticoagulant modulation of inflammation in severe sepsis. World J. Crit. Care Med..

[15] Scarlatescu E., Iba T. (2024). Deranged Balance of Hemostasis and Fibrinolysis in Disseminated Intravascular Coagulation: Assessment and Relevance in Different Clinical Settings. Anesthesiology.

[16] Page M.J., Pretorius E. (2020). A Champion of Host Defense: A Generic Large-Scale Cause for Platelet Dysfunction and Depletion in Infection. Semin. Thromb. Hemost..

[17] Iba T., Levi M. (2022). Intracellular communication and immunothrombosis in sepsis. J. Thromb. Haemost..

[18] Delabranche X., Helms J., Meziani F. (2017). Immunohaemostasis: A new view on haemostasis during sepsis. Ann. Intensiv. Care.

[19] Iba T., Helms J. (2023). The pathophysiology, diagnosis, and management of sepsis-associated disseminated intravascular coagulation. J. Intensiv. Care.

[20] Maneta E., Aivalioti E., Tual-Chalot S., Emini Veseli B., Gatsiou A., Stamatelopoulos K., Stellos K. (2023). Endothelial dysfunction and immunothrombosis in sepsis. Front. Immunol..

[21] Fiusa M.M., Carvalho-Filho M.A., Annichino-Bizzacchi J.M., De Paula E.V. (2015). Causes and consequences of coagulation activation in sepsis: An evolutionary medicine perspective. BMC Med..

[22] Engelmann B., Massberg S. (2013). Thrombosis as an intravascular effector of innate immunity. Nat. Reviews. Immunol..

[23] Ito T. (2014). PAMPs and DAMPs as triggers for DIC. J. Intensiv. Care.

[24] Iba T., Levy J.H., Maier C.L., Helms J., Umemura Y., Moore H., Othman M., Thachil J., Connors J.M., Levi M. (2025). Updated definition and scoring of disseminated intravascular coagulation in 2025: Communication from the ISTH SSC Subcommittee on Disseminated Intravascular Coagulation. J. Thromb. Haemost..

[25] Wada T., Gando S. (2024). Phenotypes of Disseminated Intravascular Coagulation. Thromb. Haemost..

[26] Longstaff C., Kolev K. (2015). Basic mechanisms and regulation of fibrinolysis. J. Thromb. Haemost..

[27] Weitz J.I., Fredenburgh J.C., Eikelboom J.W. (2017). A Test in Context: D-Dimer. J. Am. Coll. Cardiol..

[28] Pepperell D., Morel-Kopp M.-C., Ward C. (2014). Clinical Application of Fibrinolytic Assays.

[29] Mahmood N., Mihalcioiu C., Rabbani S.A. (2018). Multifaceted Role of the Urokinase-Type Plasminogen Activator (uPA) and Its Receptor (uPAR): Diagnostic, Prognostic, and Therapeutic Applications. Front. Oncol..

[30] Hack C.E. (2001). Fibrinolysis in disseminated intravascular coagulation. Semin. Thromb. Hemost..

[31] Gando S. (2013). Role of fibrinolysis in sepsis. Semin. Thromb. Hemost..

[32] Plug T., Meijers J.C. (2016). Structure-function relationships in thrombin-activatable fibrinolysis inhibitor. J. Thromb. Haemost..

[33] Suffredini A.F., Harpel P.C., Parrillo J.E. (1989). Promotion and subsequent inhibition of plasminogen activation after administration of intravenous endotoxin to normal subjects. N. Engl. J. Med..

[34] Vincent J.-L. (2001). Microvascular endothelial dysfunction: A renewed appreciation of sepsis pathophysiology. Crit. Care.

[35] Kuiper G.J., Kleinegris M.C., van Oerle R., Spronk H.M., Lance M.D., Ten Cate H., Henskens Y.M. (2016). Validation of a modified thromboelastometry approach to detect changes in fibrinolytic activity. Thromb. J..

[36] Panigada M., Sampietro F., L’Acqua C., Zacchetti L., Anzoletti M.B., Bader R., Gattinoni L., D’Angelo A. (2016). Impaired dynamics of clot formation and hypofibrinolysis in severe sepsis are coexisting and strictly related. Intensiv. Care Med..

[37] Panigada M., Zacchetti L., L’Acqua C., Cressoni M., Anzoletti M.B., Bader R., Protti A., Consonni D., D’Angelo A., Gattinoni L. (2015). Assessment of Fibrinolysis in Sepsis Patients with Urokinase Modified Thromboelastography. PLoS ONE.

[38] Koyama K., Madoiwa S., Nunomiya S., Koinuma T., Wada M., Sakata A., Ohmori T., Mimuro J., Sakata Y. (2014). Combination of thrombin-antithrombin complex, plasminogen activator inhibitor-1, and protein C activity for early identification of severe coagulopathy in initial phase of sepsis: A prospective observational study. Crit. Care.

[39] van Deventer S.J., Buller H.R., ten Cate J.W., Aarden L.A., Hack C.E., Sturk A. (1990). Experimental endotoxemia in humans: Analysis of cytokine release and coagulation, fibrinolytic, and complement pathways. Blood.

[40] Hermans P.W., Hazelzet J.A. (2005). Plasminogen activator inhibitor type 1 gene polymorphism and sepsis. Clin. Infect. Dis..

[41] Emonts M., Bruijne E.L.E.D., GuimarÃEs A.H.C., Declerck P.J., Leebeek F.W.G., Maat M.P.M.D., Rijken D.C., Hazelzet J.A., Gils A. (2008). Thrombin activatable fibrinolysis inhibitor is associated with severity and outcome of severe meningococcal infection in children. J. Thromb. Haemost..

[42] Zeerleder S., Schroeder V., Hack C.E., Kohler H.P., Wuillemin W.A. (2006). TAFI and PAI-1 levels in human sepsis. Thromb. Res..

[43] Madoiwa S. (2015). Recent advances in disseminated intravascular coagulation: Endothelial cells and fibrinolysis in sepsis-induced DIC. J. Intensiv. Care.

[44] Madoiwa S., Tanaka H., Nagahama Y., Dokai M., Kashiwakura Y., Ishiwata A., Sakata A., Yasumoto A., Ohmori T., Mimuro J. (2011). Degradation of cross-linked fibrin by leukocyte elastase as alternative pathway for plasmin-mediated fibrinolysis in sepsis-induced disseminated intravascular coagulation. Thromb. Res..

[45] Aird W.C. (2003). The role of the endothelium in severe sepsis and multiple organ dysfunction syndrome. Blood.

[46] Gando S., Kameue T., Sawamura A., Hayakawa M., Hoshino H., Kubota N. (2007). An alternative pathway for fibrinolysis is activated in patients who have undergone cardiopulmonary bypass surgery and major abdominal surgery. Thromb. Res..

[47] Gando S., Hayakawa M., Sawamura A., Hoshino H., Oshiro A., Kubota N., Jesmin S. (2007). The activation of neutrophil elastase-mediated fibrinolysis is not sufficient to overcome the fibrinolytic shutdown of disseminated intravascular coagulation associated with systemic inflammation. Thromb. Res..

[48] Bach-Gansmo E.T., Halvorsen S., Godal H.C., Skjønsberg O.H. (1995). Impaired clot lysis in the presence of human neutrophil elastase. Thromb. Res..

[49] Gould T.J., Vu T.T., Stafford A.R., Dwivedi D.J., Kim P.Y., Fox-Robichaud A.E., Weitz J.I., Liaw P.C. (2015). Cell-Free DNA Modulates Clot Structure and Impairs Fibrinolysis in Sepsis. Arter. Thromb. Vasc. Biol..

[50] Adamzik M., Eggmann M., Frey U.H., Gorlinger K., Brocker-Preuss M., Marggraf G., Saner F., Eggebrecht H., Peters J., Hartmann M. (2010). Comparison of thromboelastometry with procalcitonin, interleukin 6, and C-reactive protein as diagnostic tests for severe sepsis in critically ill adults. Crit. Care.

[51] Andersen M.G., Hvas C.L., Tonnesen E., Hvas A.M. (2014). Thromboelastometry as a supplementary tool for evaluation of hemostasis in severe sepsis and septic shock. Acta Anaesthesiol. Scand..

[52] Bouck E.G., Denorme F., Holle L.A., Middelton E.A., Blair A.M., Laat B.d., Schiffman J.D., Yost C.C., Rondina M.T., Wolberg A.S. (2021). COVID-19 and Sepsis Are Associated With Different Abnormalities in Plasma Procoagulant and Fibrinolytic Activity. Arterioscler. Thromb. Vasc. Biol..

[53] Zouaoui Boudjeltia K., Piagnerelli M., Brohée D., Guillaume M., Cauchie P., Vincent J.-L., Remacle C., Bouckaert Y., Vanhaeverbeek M. (2004). Relationship between CRP and hypofibrinolysis: Is this a possible mechanism to explain the association between CRP and outcome in critically ill patients?. Thromb. J..

[54] Brenner T., Schmidt K., Delang M., Mehrabi A., Bruckner T., Lichtenstern C., Martin E., Weigand M.A., Hofer S. (2012). Viscoelastic and aggregometric point-of-care testing in patients with septic shock-cross-links between inflammation and haemostasis. Acta Anaesthesiol. Scand..

[55] Brewer J.S., Hvas C.L., Hvas A.-M., Larsen J.B. (2024). Impaired Whole-Blood Fibrinolysis is a Predictor of Mortality in Intensive Care Patients. TH Open.

[56] Cruz D.B.D., Helms J., Aquino L.R., Stiel L., Cougourdan L., Broussard C., Chafey P., Riès-Kautt M., Meziani F., Toti F. (2019). DNA-bound elastase of neutrophil extracellular traps degrades plasminogen, reduces plasmin formation, and decreases fibrinolysis: Proof of concept in septic shock plasma. FASEB J. Off. Publ. Fed. Am. Soc. Exp. Biol..

[57] Davies G.R., Pillai S., Lawrence M., Mills G.M., Aubrey R., D’Silva L., Battle C., Williams R., Brown R., Thomas D. (2016). The effect of sepsis and its inflammatory response on mechanical clot characteristics: A prospective observational study. Intensiv. Care Med..

[58] Davies G.R., Lawrence M., Pillai S., Mills G.M., Aubrey R., Thomas D., Williams R., Morris K., Evans P.A. (2017). The effect of sepsis and septic shock on the viscoelastic properties of clot quality and mass using rotational thromboelastometry: A prospective observational study. J. Crit. Care.

[59] Hartemink K.J., Hack C.E., Groeneveld A.B. (2010). Relation between coagulation/fibrinolysis and lactate in the course of human septic shock. J. Clin. Pathol..

[60] Hayakawa M., Sawamura A., Gando S., Jesmin S., Naito S., Ieko M. (2012). A low TAFI activity and insufficient activation of fibrinolysis by both plasmin and neutrophil elastase promote organ dysfunction in disseminated intravascular coagulation associated with sepsis. Thromb. Res..

[61] Helling H., Schenk H.J., Pindur G., Weinrich M., Wagner B., Stephan B. (2010). Fibrinolytic and procoagulant activity in septic and haemorrhagic shock. Clin. Hemorheol. Microcirc..

[62] Hesselvik J.F., Blomback M., Brodin B., Maller R. (1989). Coagulation, fibrinolysis, and kallikrein systems in sepsis: Relation to outcome. Crit Care Med..

[63] Hoppensteadt D., Tsuruta K., Hirman J., Kaul I., Osawa Y., Fareed J. (2015). Dysregulation of Inflammatory and Hemostatic Markers in Sepsis and Suspected Disseminated Intravascular Coagulation. Clin. Appl. Thromb. Hemost..

[64] Hoshino K., Kitamura T., Nakamura Y., Irie Y., Matsumoto N., Kawano Y., Ishikura H. (2017). Usefulness of plasminogen activator inhibitor-1 as a predictive marker of mortality in sepsis. J. Intensiv. Care.

[65] Hoshino K., Nakashio M., Maruyama J., Irie Y., Kawano Y., Ishikura H. (2020). Validating plasminogen activator inhibitor-1 as a poor prognostic factor in sepsis. Acute Med. Surg..

[66] Iba T., Kidokoro A., Fukunaga M., Sugiyama K., Sawada T., Kato H. (2005). Association between the Severity of Sepsis and the Changes in Hemostatic Molecular Markers and Vascular Endothelial Damage Markers. Shock.

[67] Johansson P.I., Haase N., Perner A., Ostrowski S.R. (2014). Association between sympathoadrenal activation, fibrinolysis, and endothelial damage in septic patients: A prospective study. J. Crit. Care.

[68] Koami H., Sakamoto Y., Ohta M., Goto A., Narumi S., Imahase H., Yahata M., Miike T., Iwamura T., Yamada K.C. (2015). Can rotational thromboelastometry predict septic disseminated intravascular coagulation?. Blood Coagul. Fibrinolysis Int. J. Haemost. Thromb..

[69] Koami H., Sakamoto Y., Hirota Y., Sasaki A., Ogawa H., Furukawa Y., Matsuoka A., Shinada K., Nakayama K., Sakurai R. (2025). Effect of hypofibrinolysis on clinical outcomes of patients with septic disseminated intravascular coagulation. Thromb. Res..

[70] Koh G.C., Meijers J.C., Maude R.R., Limmathurotsakul D., Day N.P., Peacock S.J., van der Poll T., Wiersinga W.J. (2011). Diabetes does not influence activation of coagulation, fibrinolysis or anticoagulant pathways in Gram-negative sepsis (melioidosis). Thromb. Haemost..

[71] Larsen J.B., Aggerbeck M.A., Larsen K.M., Hvas C.L., Hvas A.-M. (2021). Fibrin Network Formation and Lysis in Septic Shock Patients. Int. J. Mol. Sci..

[72] Lorente J.A., García-Frade L.J., Landín L., de Pablo R., Torrado C., Renes E., García-Avello A. (1993). Time Course of Hemostatic Abnormalities in Sepsis and its Relation to Outcome. Chest.

[73] Madoiwa S., Nunomiya S., Ono T., Shintani Y., Ohmori T., Mimuro J., Sakata Y. (2006). Plasminogen activator inhibitor 1 promotes a poor prognosis in sepsis-induced disseminated intravascular coagulation. Int. J. Hematol..

[74] Massignon D., Lepape A., Bienvenu J., Barbier Y., Boileau C., Coeur P. (1994). Coagulation/fibrinolysis balance in septic shock related to cytokines and clinical state. Haemostasis.

[75] Mauri T., Bellani G., Patroniti N., Coppadoro A., Peri G., Cuccovillo I., Cugno M., Iapichino G., Gattinoni L., Pesenti A. (2010). Persisting high levels of plasma pentraxin 3 over the first days after severe sepsis and septic shock onset are associated with mortality. Intensiv. Care Med..

[76] Mavrommatis A.C., Theodoridis T., Economou M., Kotanidou A., El Ali M., Christopoulou-Kokkinou V., Zakynthinos S.G. (2001). Activation of the fibrinolytic system and utilization of the coagulation inhibitors in sepsis: Comparison with severe sepsis and septic shock. Intensiv. Care Med..

[77] Mei H., Jiang Y., Luo L., Huang R., Su L., Hou M., Wang X., Deng J., Hu Y. (2019). Evaluation the combined diagnostic value of TAT, PIC, tPAIC, and sTM in disseminated intravascular coagulation: A multi-center prospective observational study. Thromb. Res..

[78] Müller M.C.A., Meijers J.C., van Meenen D.M., Thachil J., Juffermans N.P. (2019). Thromboelastometry in critically ill patients with disseminated intravascular coagulation. Blood Coagul. Fibrinolysis Int. J. Haemost. Thromb..

[79] Park K.J., Kim H.J., Hwang S.C., Lee S.M., Lee Y.H., Hahn M.H., Kim S.K., Lee W.Y. (1999). The imbalance between coagulation and fibrinolysis is related to the severity of the illness and the prognosis in sepsis. Korean J. Intern. Med..

[80] Park R., Song J., An S.S. (2010). Elevated levels of activated and inactivated thrombin-activatable fibrinolysis inhibitor in patients with sepsis. Korean J. Hematol..

[81] Prakash S., Verghese S., Roxby D., Dixon D., Bihari S., Bersten A. (2015). Changes in fibrinolysis and severity of organ failure in sepsis: A prospective observational study using point-of-care test--ROTEM. J. Crit. Care.

[82] Sanches L.C., Pontes Azevedo L.C., Salomão R., Noguti M.A., Brunialti M., Lourenço D.M., Machado F.R. (2014). Association between early glycemic control and improvements in markers of coagulation and fibrinolysis in patients with septic shock–induced stress hyperglycemia. J. Crit. Care.

[83] Savioli M., Cugno M., Polli F., Taccone P., Bellani G., Spanu P., Pesenti A., Iapichino G., Gattinoni L. (2009). Tight glycemic control may favor fibrinolysis in patients with sepsis. Crit. Care Med..

[84] Scarlatescu E., Lance M.D., White N.J., Tomescu D.R. (2018). Thromboelastometric prediction of mortality using the kinetics of clot growth in critically ill septic patients. Blood Coagul. Fibrinolysis Int. J. Haemost. Thromb..

[85] Scarlatescu E., White N.J., Tomescu D.R. (2020). Standard and derived rotational thromboelastometry parameters for prediction of disseminated intravascular coagulation in septic patients. Blood Coagul. Fibrinolysis Int. J. Haemost. Thromb..

[86] Schmitt F.C.F., Manolov V., Morgenstern J., Fleming T., Heitmeier S., Uhle F., Al-Saeedi M., Hackert T., Bruckner T., Schöchl H. (2019). Acute fibrinolysis shutdown occurs early in septic shock and is associated with increased morbidity and mortality: Results of an observational pilot study. Ann. Intensiv. Care.

[87] Semeraro F., Colucci M., Caironi P., Masson S., Ammollo C.T., Teli R., Semeraro N., Magnoli M., Salati G., Isetta M. (2017). Platelet Drop and Fibrinolytic Shutdown in Patients With Sepsis. Crit. Care Med..

[88] Semeraro F., Ammollo C., Caironi P., Masson S., Latini R., Panigada M., Semeraro N., Gattinoni L., Colucci M. (2018). Low D-dimer levels in sepsis: Good or bad?. Thromb Res..

[89] Semeraro F., Ammollo C.T., Caironi P., Masson S., Latini R., Panigada M., Pesenti A., Semeraro N., Gattinoni L., Colucci M. (2020). D-dimer corrected for thrombin and plasmin generation is a strong predictor of mortality in patients with sepsis. Blood Transfus..

[90] Shaw A.D., Vail G.M., Haney D.J., Xie J., Williams M.D. (2011). Severe protein C deficiency is associated with organ dysfunction in patients with severe sepsis. J. Crit. Care.

[91] Sivula M., Pettila V., Niemi T.T., Varpula M., Kuitunen A.H. (2009). Thromboelastometry in patients with severe sepsis and disseminated intravascular coagulation. Blood Coagul. Fibrinolysis Int. J. Haemost. Thromb..

[92] Wiersinga W.J., Meijers J.C., Levi M., Van ‘t Veer C., Day N.P., Peacock S.J., van der Poll T. (2008). Activation of coagulation with concurrent impairment of anticoagulant mechanisms correlates with a poor outcome in severe melioidosis. J. Thromb. Haemost..

[93] Zheng Z., Mukhametova L., Boffa M.B., Moore E.E., Wolberg A.S., Urano T., Kim P.Y. (2023). Assays to quantify fibrinolysis: Strengths and limitations. Communication from the International Society on Thrombosis and Haemostasis Scientific and Standardization Committee on fibrinolysis. J. Thromb. Haemost..

[94] Raaphorst J., Johan Groeneveld A.B., Bossink A.W., Erik Hack C. (2001). Early inhibition of activated fibrinolysis predicts microbial infection, shock and mortality in febrile medical patients. Thromb. Haemost..

[95] Mesters R.M., Florke N., Ostermann H., Kienast J. (1996). Increase of plasminogen activator inhibitor levels predicts outcome of leukocytopenic patients with sepsis. Thromb. Haemost..

[96] Tipoe T.L., Wu W.K.K., Chung L., Gong M., Dong M., Liu T., Roever L., Ho J., Wong M.C.S., Chan M.T.V. (2018). Plasminogen Activator Inhibitor 1 for Predicting Sepsis Severity and Mortality Outcomes: A Systematic Review and Meta-Analysis. Front. Immunol..

[97] Miszta A., Huskens D., Donkervoort D., Roberts M.J.M., Wolberg A.S. (2021). Assessing Plasmin Generation in Health and Disease. Int. J. Mol. Sci..

[98] Rijken D.C., Hoegee-De Nobel E., Jie A.F.H., Atsma D.E., Schalij M.J., Nieuwenhuizen W. (2008). Development of a new test for the global fibrinolytic capacity in whole blood. J. Thromb. Haemost..

[99] Scarlatescu E., Lance M.D., White N.J., Arama S.S., Tomescu D.R. (2018). Effects of malignancy on blood coagulation in septic intensive care patients. Blood Coagul. Fibrinolysis Int. J. Haemost. Thromb..

[100] Raza I., Davenport R., Rourke C., Platton S., Manson J., Spoors C., Khan S., De’Ath H.D., Allard S., Hart D.P. (2013). The incidence and magnitude of fibrinolytic activation in trauma patients. J. Thromb. Haemost. JTH.

[101] Coupland L.A., Rabbolini D.J., Schoenecker J.G., Crispin P.J., Miller J.J., Ghent T., Medcalf R.L., Aneman A.E. (2023). Point-of-care diagnosis and monitoring of fibrinolysis resistance in the critically ill: Results from a feasibility study. Crit. Care.

[102] Evans P.A., Hawkins K., Morris R.H., Thirumalai N., Munro R., Wakeman L., Lawrence M.J., Williams P.R. (2010). Gel point and fractal microstructure of incipient blood clots are significant new markers of hemostasis for healthy and anticoagulated blood. Blood.

